# Mobility Change and COVID-19 in Japan: Mobile Data Analysis of Locations of Infection

**DOI:** 10.2188/jea.JE20200625

**Published:** 2021-06-05

**Authors:** Shohei Nagata, Tomoki Nakaya, Yu Adachi, Toru Inamori, Kazuto Nakamura, Dai Arima, Hiroshi Nishiura

**Affiliations:** 1 Graduate School of Environmental Studies, Tohoku University, Sendai, Japan; 2 ALBERT Inc, Tokyo, Japan; 3 Graduate School of Medicine and Faculty of Medicine, Kyoto University, Kyoto, Japan

**Keywords:** communicable diseases, COVID-19, big data, mobility, Japan

## Abstract

**Background:**

As the COVID-19 pandemic spread, the Japanese government declared a state of emergency on April 7, 2020 for seven prefectures, and on April 16, 2020 for all prefectures. The Japanese Prime Minister and governors requested people to adopt self-restraint behaviors, including working from home and refraining from visiting nightlife spots. However, the effectiveness of the mobility change due to such requests in reducing the spread of COVID-19 has been little investigated. The present study examined the association of the mobility change in working, nightlife, and residential places and the COVID-19 outbreaks in Tokyo, Osaka, and Nagoya metropolitan areas in Japan.

**Methods:**

First, we calculated the daily mobility change in working, nightlife, and residential places compared to the mobility before the outbreak using mobile device data. Second, we estimated the sensitivity of mobility changes to the reproduction number by generalized least squares.

**Results:**

Mobility change had already started in March, 2020. However, mobility reduction in nightlife places was particularly significant due to the state of emergency declaration. Although the mobility in each place type was associated with the COVID-19 outbreak, the mobility changes in nightlife places were more significantly associated with the outbreak than those in the other place types. There were regional differences in intensity of sensitivity among each metropolitan area.

**Conclusions:**

Our findings indicated the effectiveness of the mobility changes, particularly in nightlife places, in reducing the outbreak of COVID-19.

## INTRODUCTION

**Figure 1.**
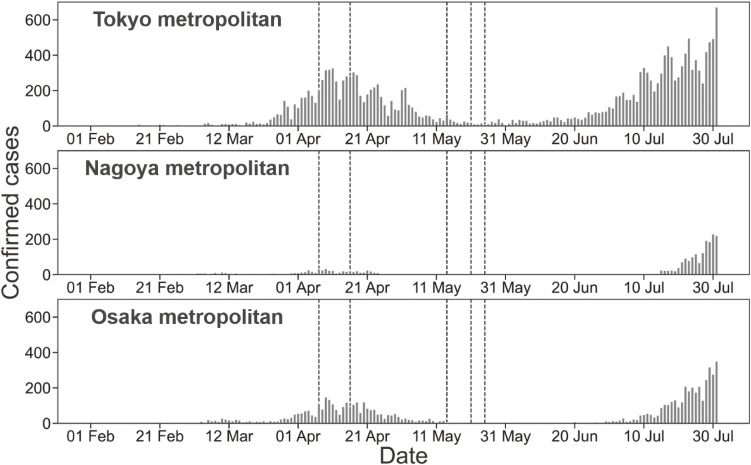
Number of daily confirmed cases in major metropolitan areas. Vertical dash lines represent the start/end date of state of emergency (SOE) declaration. (On April 7, 2020, SOE declaration for Tokyo, Osaka, Kanagawa, Saitama, Chiba, Hyogo, and Fukuoka; on April 16, 2020, SOE declaration for the remaining prefectures; on May 14, 2020, lifting of SOE declaration for prefectures excluding the ones in Hokkaido, Tokyo, and Osaka metropolitan areas; on May 21, 2020, lifting of SOE declaration for Kyoto, Osaka, and Hyogo; on May 25, 2020, lifting of SOE declaration for Hokkaido, Saitama, Chiba, Tokyo, and Kanagawa.)

With the global spread of the novel coronavirus disease (COVID-19), many countries have implemented non-pharmaceutical interventions, including lockdowns and travel restrictions, to control the pandemic.^1^^,^^2^ In Japan, the government declared a state of emergency (SOE) on April 7, 2020 for seven prefectures, where the confirmed cases had increased markedly (Figure 1), and requested people to adopt self-restraining behaviors, such as cancelling nonessential outings and avoiding the “3Cs” conditions (closed spaces, crowded places, and close-contact settings).^3^ On April 11, the Prime Minister urged people to work from home,^4^ and governors advised people to refrain from visiting nightlife spots, such as nightclubs and bars. SOE was applied to all 47 prefectures on April 16.^3^ To understand contact patterns while such social measures were in place, published studies, including one from Japan, reported that human mobility was associated with the incidence of COVID-19.^5^^–^^11^ Despite numerous studies, the effectiveness of reducing contact in focal areas at high risk (eg, workplaces or nightlife places) is yet to be understood. The present study aimed to examine the mobility changes in work, nightlife, and residential places in Japan based on mobile device data and to clarify the association of mobility change at these places with COVID-19 incidences.

## METHODS

We observed the mobility changes resulting from the outbreak of COVID-19 in three major metropolitan areas—Tokyo, Osaka, and Nagoya—from March 1 to July 31, 2020. Although there are multiple definitions of metropolitan areas in Japan, in this study, Tokyo, Kanagawa, Saitama, and Chiba Prefectures were defined as the Tokyo metropolitan area; Osaka, Kyoto, Hyogo, Shiga, Nara, and Wakayama Prefectures were defined as the Osaka metropolitan area; and Aichi, Gifu, and Mie Prefectures were defined as the Nagoya metropolitan area.

### Mobility data and defining the specific places

“Mobile Spatial Statistics” (DOCOMO InsightMarketing, Inc., Tokyo, Japan), which provide the estimated hourly population in a 500-m-square grid based on mobile device locations,^12^ were employed as the mobility data. We classified the grids into work, nightlife, or residential place based on the median of population at specific times (midnight: 3:00 AM–5:59 AM, daytime: 2:00 PM–4:59 PM, nighttime: 8:00 PM–10:59 PM) of each weekday from January 3 to February 6, 2020. When the median of daytime population was 10,000 or more and twice as large as the median of midnight population, the corresponding grid was designated as a “workplace”. When the median of nighttime population was 10,000 or more and twice as large as the median of midnight population, the grid was designated as a “nightlife place”. When the median of midnight population was 100 or more and smaller than the median of daytime population, the grid was set as a “residential place”.

### Mobility change index

For the mobility change index of each place type, we determined the ratio of the daily population to the baseline population in each grid from March to July 2020. Specifically, the mobility change index *m* of each specific place type *p* on day *t* is as follows:

$$m_{p,t}=\frac{{\text{Pop}}_{p,t}}{B_{p}},$$

where *Pop_p_*_,_*_t_* is the total hourly population in *p* at specific times (workplaces: 2:00 PM–4:59 PM; nightlife places: 8:00 PM–10:59 PM; residential places: 3:00 AM–5:59 AM) on day *t*. The baseline in each place type (*B_p_*) was defined according to the median of the total hourly population at the corresponding time on a day, from January 3 to February 6, 2020. In the statistical analysis described below, we employed a 7-day moving average to exclude the day-of-the-week effects on the daily mobility change index, *m*. The moving average *M* of each specific place type *p* on the day *t* is as follows:

$$M_{p,t}=\frac{\sum_{i=t-6}^{t}m_{p,i}}{7}.$$

### Epidemiological data

For obtaining the daily counts of positively confirmed cases, we used the open-source epidemiological data provided by J.A.G JAPAN Corp,^13^ which summarizes the press releases of confirmed cases published by local governments. We counted the number of daily positive cases, excluding re-positive cases, by each metropolitan area based on the confirmed date (or reported date if confirmed date of the case is unknown).

### Statistical analysis

To evaluate the relationship between mobility changes and COVID-19 incidences, we employed the following model (model 1) to predict the cumulative number of confirmed cases in the last 7 days beginning from day *t*, *y_t_*:

$$y_{t}=\alpha +\beta_{t}y_{t-7}+\varepsilon_{t},$$

$$\beta_{t}=\beta_{0}+\beta_{1}M_{p,t-L},$$

$$\varepsilon_{t}=\rho \varepsilon_{t-1}+\omega_{t},$$

where *α*, *β*_0_, *β*_1_, and *ρ* are parameters to be estimated through generalized least squares. The response variable, *y_t_*, is defined as:

$$y_{t}=\sum_{i=t-6}^{t}c_{i},$$

where *c_i_* is the number of daily confirmed cases on day *i*. In the model, the coefficient, *β_t_*, is the increasing rate of the 7-day cumulative cases, *y_t_*, compared to the earlier 7 days ($y_{t-7}=\sum_{i=t-13}^{t-7}c_{i}$). This parameter approximately represents the number of people an infected person infects on day *t*. We assumed that *β_t_* is dependent on the mobility change index of the specific place *p* at day *t* with *L* day lag, *M_p_*_,_*_t_*_−_*_L_*. Because the mobility changes may be associated with the incidence after around 2 weeks, due to the time lag between infection and diagnosis or reporting,^5^ we substituted the value from 7 to 20 for the lag period, *L*, and selected the optimal value of *L* to estimate *β_t_* based on the Akaike Information Criterion (AIC) by each specific place type and metropolitan area. In addition, with the independent and identically distributed white noise, *ω_t_* ∼ *_iid_N*(0,*σ*^2^), we used the first-order autoregressive error, *ε_t_*, to adjust the temporal dependency caused by unknown factors, where *ρ* is the so-called autocorrelation parameter; *E*(*ε_t_*) = 0, *Var*(*ε_t_*) = *σ*^2^/(1 − *ρ*^2^), and *Cov*(*ε_t_*, *ε_t_*_−1_) = *ρσ*^2^/(1 − *ρ*^2^).

We also considered the model in which *β_t_* is simultaneously dependent on the mobility change indices of all place types to improve the predictive accuracy of the model (model 2). In this model, *β_t_* is as follows:

$$\beta_{t}=\beta_{0}+\sum_{p\in \{\text{Work},\text{Nightlife},\text{Residential}\}}\beta_{p}M_{p,t-L_{p}}.$$

Regarding the lag period of each place category, *p* (*L_p_*), we substituted the optimal value for each place type determined through the results of model 1 in the respective metropolitan areas.

## RESULTS

**Figure 2.**
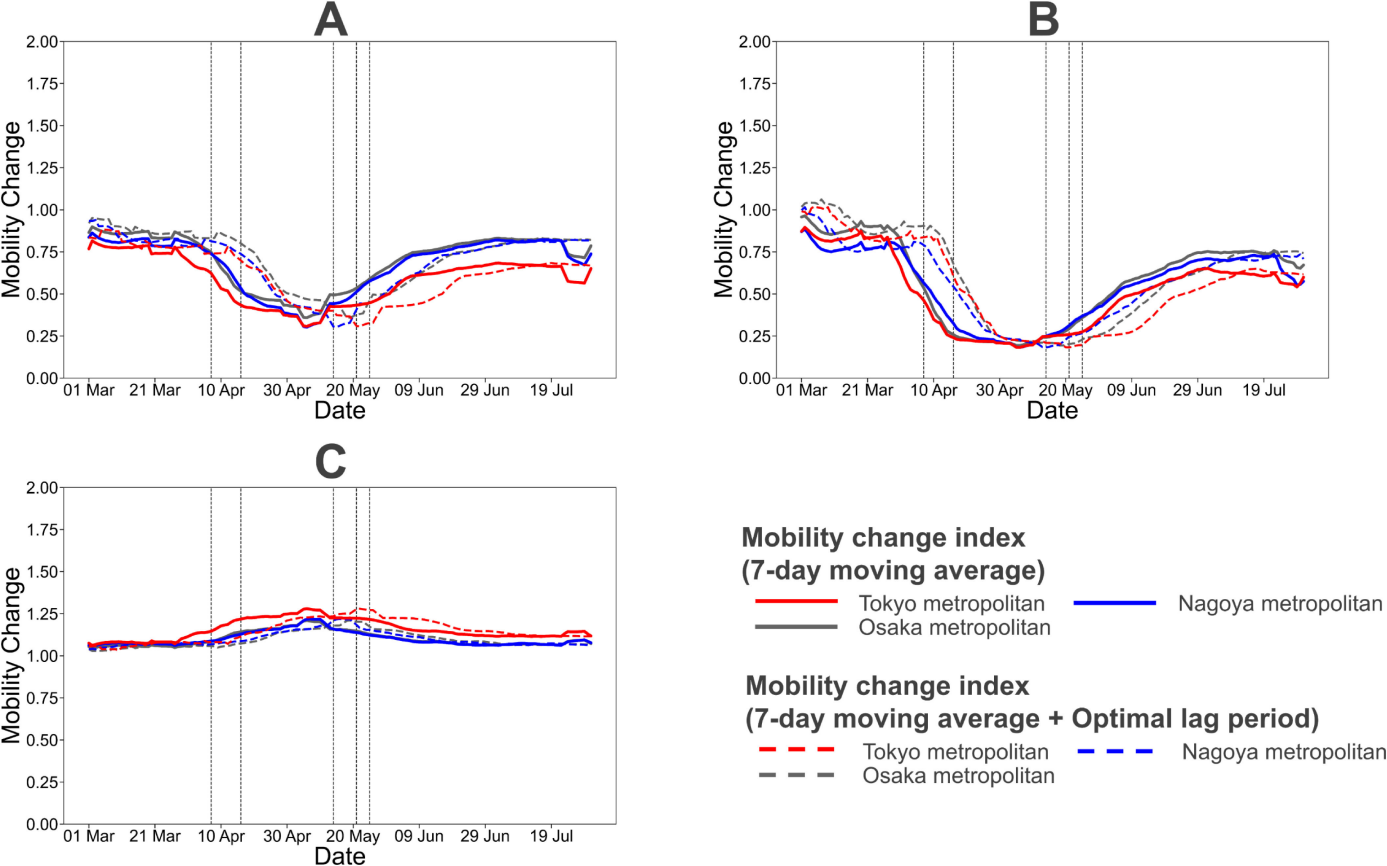
Daily change of mobility in workplaces (A), nightlife places (B), and residential places (C). The solid and dashed lines represent the 7-day moving average of the mobility change index and the 7-day moving average of the mobility change index delayed by the optimal lag period, respectively. The mobility change index is represented by the ratio of the population of each place at specific times (workplaces: 2:00 PM–4:59 PM, nightlife places: 8:00 PM–10:59 PM, residential places: 3:00 AM–5:59 AM) to the baseline population. The baseline in each place was defined based on the median of population at corresponding time of each day from January 3, 2020 to February 6, 2020. The optimal lag period was determined using AIC. Vertical dash lines represent the start/end date of state of emergency (SOE) declaration. (On April 7, 2020, SOE declaration for Tokyo, Osaka, Kanagawa, Saitama, Chiba, Hyogo, and Fukuoka; on April 16, 2020, SOE declaration for the remaining prefectures; on May 14, 2020, lifting of SOE declaration for prefectures excluding the ones in Hokkaido, Tokyo, and Osaka metropolitan areas; on May 21, 2020, lifting of SOE declaration for Kyoto, Osaka, and Hyogo; on May 25, 2020, lifting of SOE declaration for Hokkaido, Saitama, Chiba, Tokyo, and Kanagawa.)

Although the mobility change indices in workplaces and nightlife places had already declined slightly as of early March, it showed a drastic downward trend in all regions from late March, before SOE was declared (Figure 2A and Figure 2B). Because mobility reduction in nightlife places was particularly significant during SOE, it can be presumed that the majority of people refrained from visiting nightlife spots, as requested by the government. In contrast, the index in residential places that reflected staying at home showed an upward trend from March to May (Figure 2C).

According to the estimated *β*_1_ and the optimal lag period of the models with the single mobility change index (model 1), the mobility changes in workplaces and nightlife places were positively associated with the incidence after 8 to 16 days in all metropolitan areas, and the residential places’ mobility was negatively associated (Table 1); that is, the decrease in people visiting workplaces or nightlife places, as well as the increase in stay-at-home population, could have led to reducing the outbreak, although its sensitivity varied by area type. Notably, judging from Nagelkerke’s R^2^, the mobility changes in the nightlife spots better explained the outbreak of COVID-19 compared to workplaces and residential areas in the respective metropolitan areas. However, the goodness of fit of the models based on the Tokyo metropolitan area’s mobility changes was lower than that of the other areas’ models.

**Table 1.** Estimated *β_t_* of Model 1

Area	Place	Lag period	AIC	R^2^	*β*_0_			*β*_1_		
	
					Coef.	95% CI	*P*	Coef.	95% CI	*P*
Tokyo metropolitan	Workplace	16	1,706.30	0.25	−0.91	−1.43 to −0.39	<0.001^***^	2.06	1.27 to 2.84	<0.001^***^
	Nightlife place	15	1,697.88	0.29	−0.37	−0.66 to −0.08	0.012^*^	1.55	1.05 to 2.06	<0.001^***^
	Residential place	16	1,705.05	0.25	6.31	4.10 to 8.52	<0.001^***^	−5.20	−7.13 to −3.27	<0.001^***^

Nagoya metropolitan	Workplace	9	1,253.60	0.72	−2.50	−3.70 to −1.31	<0.001^***^	5.23	3.72 to 6.75	<0.001^***^
	Nightlife place	8	1,238.71	0.75	−1.07	−1.74 to −0.41	0.002^**^	4.11	3.11 to 5.10	<0.001^***^
	Residential place	9	1,249.40	0.73	26.05	19.35 to 32.75	<0.001^***^	−22.86	−29.12 to −16.60	<0.001^***^

Osaka metropolitan	Workplace	13	1,436.46	0.54	−1.01	−1.52 to −0.50	<0.001^***^	2.41	1.74 to 3.08	<0.001^***^
	Nightlife place	13	1,426.41	0.57	−0.22	−0.49 to 0.06	0.119	1.70	1.28 to 2.12	<0.001^***^
	Residential place	13	1,435.25	0.54	10.99	8.19 to 13.78	<0.001^***^	−9.42	−11.99 to −6.84	<0.001^***^

AIC, Akaike Information Criterion; CI, confidence interval.

^*^Coef.: Coefficient, R2: Nagelkerke’s R2 with the null model assuming zero coefficients of explanatory variables with AR1 error, “^***^”, “^**^”, and “^*^”, denote the statistical significance at 0.1%, 1%, and 5% levels, respectively. The sample size for each model was 153.

In addition, while the models with all mobility change variables (model 2) slightly improved the accuracy of predicting the number of new positive cases, their results also indicate that the mobility changes in nightlife places were more significantly associated with the outbreak than those in workplaces and residential places in each metropolitan area (Table 2). Model 2 also shows that the relationships of the residential places’ mobility changes to the outbreak were statistically significant only in the Nagoya metropolitan area, and the mobility changes in workplaces were no longer significantly associated with the outbreak in all metropolitan areas.

**Table 2.** Estimated *β_t_* of Model 2

Area	AIC	R^2^				Workplaces			Nightlife places			Residential places		
			*β*_0_			*β_Work_*			*β_Nightlife_*			*β_Residential_*		
			
			Coef.	95% CI	*P*	Coef.	95% CI	*P*	Coef.	95% CI	*P*	Coef.	95% CI	*P*
Tokyo metropolitan	1,699.55	0.30	0.69	−9.78 to 11.17	0.896	0.44	−2.50 to 3.39	0.766	1.15	0.41 to 1.89	0.003^**^	−1.00	−8.58 to 6.57	0.794
Nagoya metropolitan	1,238.50	0.76	22.70	−1.36 to 46.76	0.064	−3.63	−8.69 to 1.42	0.158	3.65	1.80 to 5.51	<0.001^***^	−19.28	−38.56 to 0.00	0.050^*^
Osaka metropolitan	1,427.24	0.58	9.95	−1.54 to 21.44	0.089	−2.37	−5.36 to 0.62	0.119	1.96	0.82 to 3.10	<0.001^***^	−7.90	−16.89 to 1.09	0.085

AIC, Akaike Information Criterion; and CI, confidence interval.

^*^Coef.: Coefficient, R2: Nagelkerke’s R2 with the null model assuming zero coefficients of explanatory variables with AR1 error, “^***^”, “^**^”, and “^*^”, denote the statistical significance at the 0.1%, 1%, and 5% levels, respectively. The sample size for each model was 153.

## DISCUSSION

We demonstrated that the mobility changes in all types of places were associated with COVID-19 incidence in Japan. Although the mobility had been slightly reduced in March, possibly reflecting the increased individual awareness of infection prevention, mobility in nightlife places was clearly reduced from mid-April to mid-May and was more strongly associated with the trends of confirmed cases compared to other potential locations of transmission. In fact, a published study has shown that the proportion of positive cases in the nightlife group was significantly higher than that in the non-nightlife group based on the SARS-CoV-2 PCR test at a clinic in Tokyo from early March to late April.^14^ Our finding and such published evidence imply that SOE and public warnings to avoid nightlife places were effective in reducing the outbreak.

Regarding the regional differences in the relationship between the mobility change and outbreak, the sensitivity and predictive accuracy of mobility in the Tokyo metropolitan area to new positive cases was the lowest in the results of models with a single mobility change (model 1). Moreover, the models with all mobility change variables (model 2) showed that the mobility changes in residential places were slightly related to the outbreak only in the Nagoya metropolitan area, but those in the Tokyo and Osaka metropolitan areas were not. These findings suggest that there may still be various opportunities having risks of infection (such as a nosocomial infection, infection at jobs where face-to-face interaction is required, or infections at daily life areas near residential places) in largely populated regions, such as the Tokyo and Osaka metropolitan areas, even though mobility was reduced in highly-sensitive places, and the stay-at-home population increased.

Our study has several limitations. First, we used the confirmed date to compile the incidence data, because several local governments have not disclosed their onset date, but ideally the date of illness onset would more properly reflect the epidemic dynamics. The differences in the optimal lag periods among the metropolitan areas could possibly be explained by regional differences in the period from onset to diagnosis/reporting, due to the testing system. Second, the choice of “place” we analyzed was defined only by the ratio of population during a specific time, and there is a possibility that some places might have been wrongly identified as nightlife places. However, we manually ascertained that the primary business/nightlife districts are correctly included in workplaces/nightlife places that we defined (eMaterials 1). Third, our study considered only the mobility changes as an environmental factor that was associated with the outbreak of COVID-19. Further studies are needed to ascertain the effect of mobility changes on the outbreak by taking into account other environmental factors, such as improvements in testing and treatment systems, seasonal effects, and increase in individual awareness of infection prevention.

Our study’s findings can be used for designing future public health and social measures against COVID-19. The results indicate that mobility reduction, particularly in nightlife places, may contribute to reducing the transmission of infectious diseases. This will help in implementing mobility reduction at appropriate places in the future.

## APPENDIX A. SUPPLEMENTARY DATA

eMaterials 1. Geographical distribution of workplaces and nightlife-places
