# Supplementary material for: Mobility Change and COVID-19 in Japan: Mobile Data Analysis of Locations of Infection
Source: J Epidemiol. 2021 Jun 5;31(6):387–91. doi: 10.2188/jea.JE20200625 (PMC8126677; doi:10.2188/jea.JE20200625)
Supplement: Supplementary file 1 [file je-31-387-s001.pdf]

eMaterials 1. Geographical distribution of workplaces and nightlife-places

To ascertain the validity of the workplaces/nightlife-places defined, we manually compared the geographical distribution of the primary business/nightlife districts in three major metropolitan areas. This document shows the degree of distribution overlaps through thematic maps.

Workplaces

For comparison with the business districts as concentrations of working people, we identified 500 m square grids where there was a large working population — in central Tokyo, Nagoya city, and Osaka city — based on the 2014 Economic Census. When the number of employees was equal to or larger than its mean plus one standard deviation, the grid was herewith defined as a primary business district. The following maps show the primary business districts and the workplaces layers overlaid for the three cities (**eMaterials Figure 1**). The solid green areas that we defined widely match the red-hatched areas, especially in central Tokyo.

Nightlife-places

The list of primary nightlife spots is available for central Tokyo, Osaka city, and Nagoya city on their respective police department (PD) websites.[1–3] The following maps visualize the nightlife spots listed by PD (indicated in red points or hatched areas) and the nightlife places that we defined (indicated in the solid blue area) (**eMaterials Figure 2**). The primary nightlife spots, except for Asakusa and Sugamo/Otsuka in Tokyo, and Imaike in Nagoya, include the nightlife places we defined.

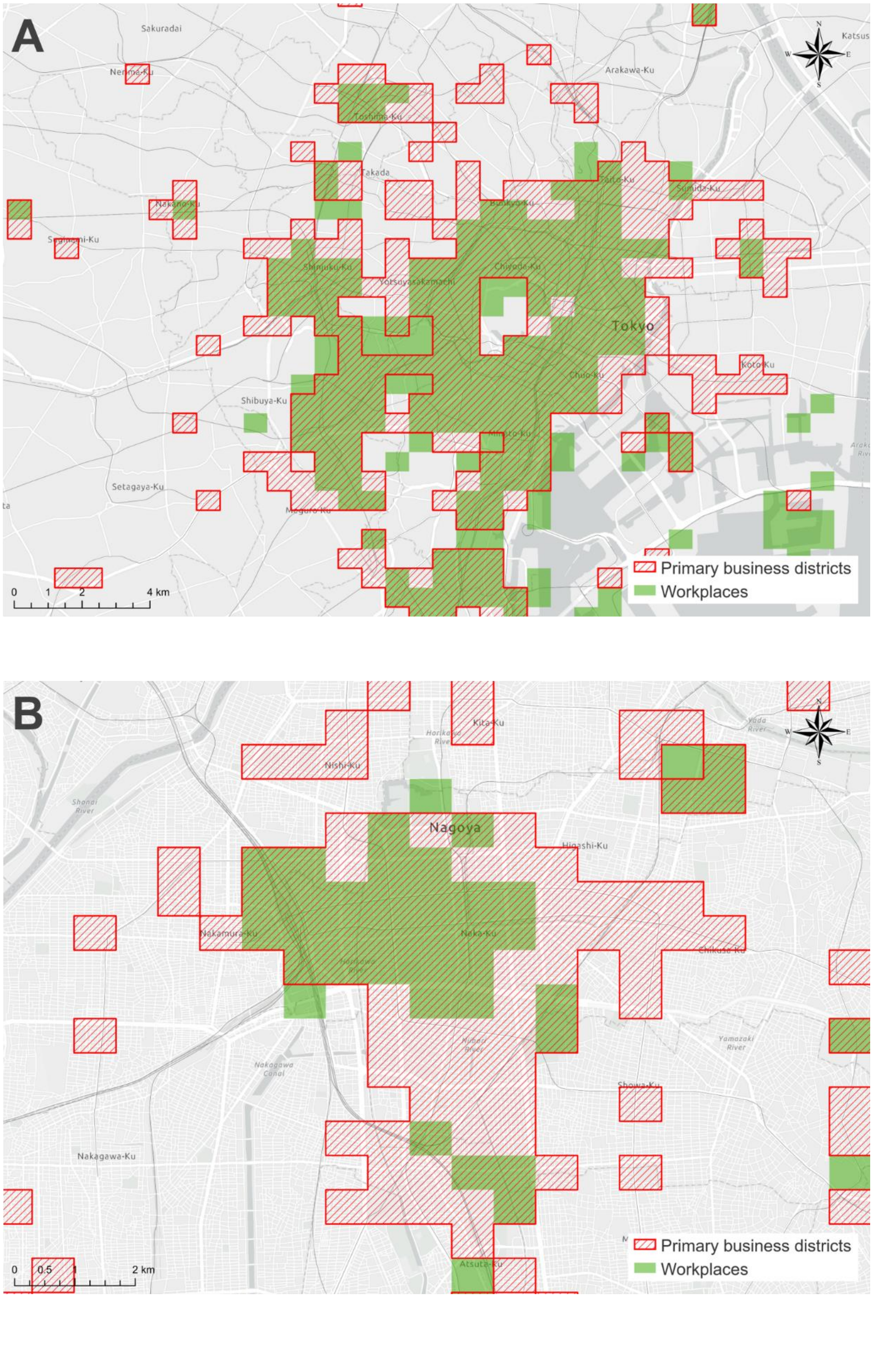
A
Sakuradai
Nerima-Ku
Arakawa-Ku
Toshima-Ku
Takada
Taito-Ku
Sumida-Ku
Nakano-Ku
Bunkyo-Ku
Shinjuku-Ku
Chiyoda-Ku
Tokyo
Koto-Ku
Chuo-Ku
Shibuya-Ku
Setagaya-Ku
Meguro-Ku
Primary business districts
Workplaces
0 1 2 4 km
B
Kita-Ku
Nishi-Ku
Nagoya
Higashi-Ku
Nakamura-Ku
Naka-Ku
Chikusa-Ku
Nakagawa Canal
Showa-Ku
Nakagawa-Ku
Atsuta-Ku
Primary business districts
Workplaces
0 0.5 1 2 km

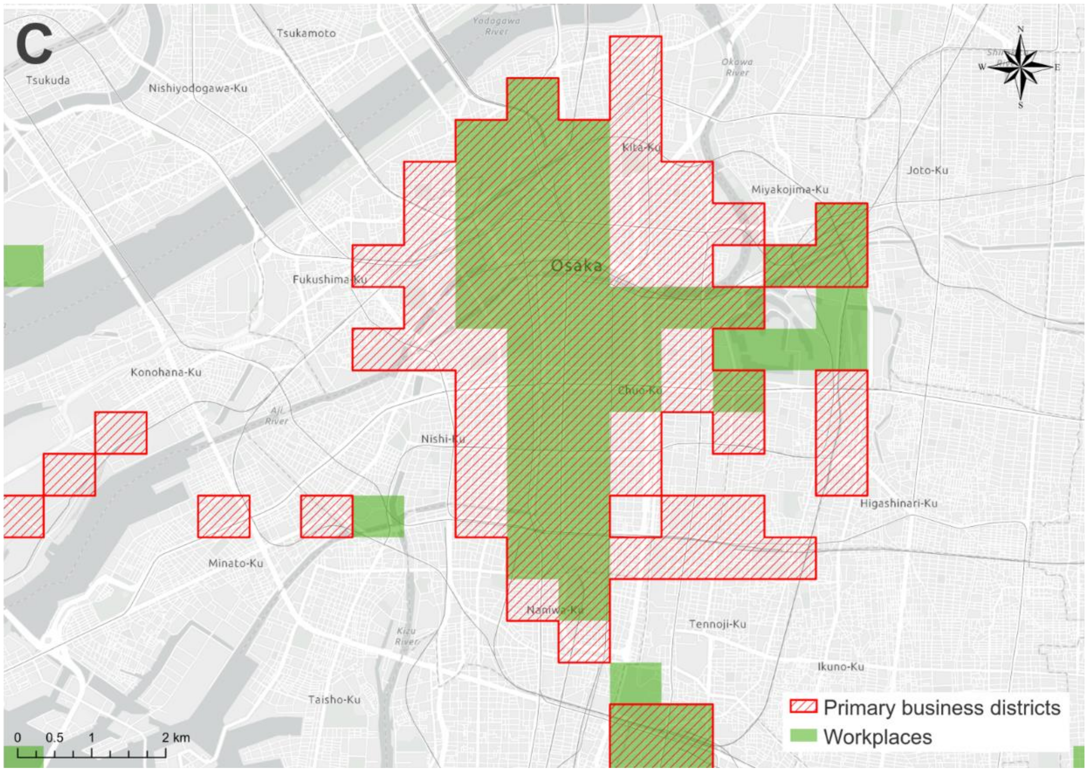

eMaterials Figure 1. Geographical distribution of the primary business districts and workplaces in Central Tokyo (A), Nagoya city (B), and Osaka city (C). Solid green areas represent the workplaces we defined. Red-hatched areas represent the primary business districts based on the 2014 Economic Census. Map sources: Esri, HERE, Garmin, FAO, NOAA, USGS, (c) OpenStreetMap contributors, and the GIS User Community.

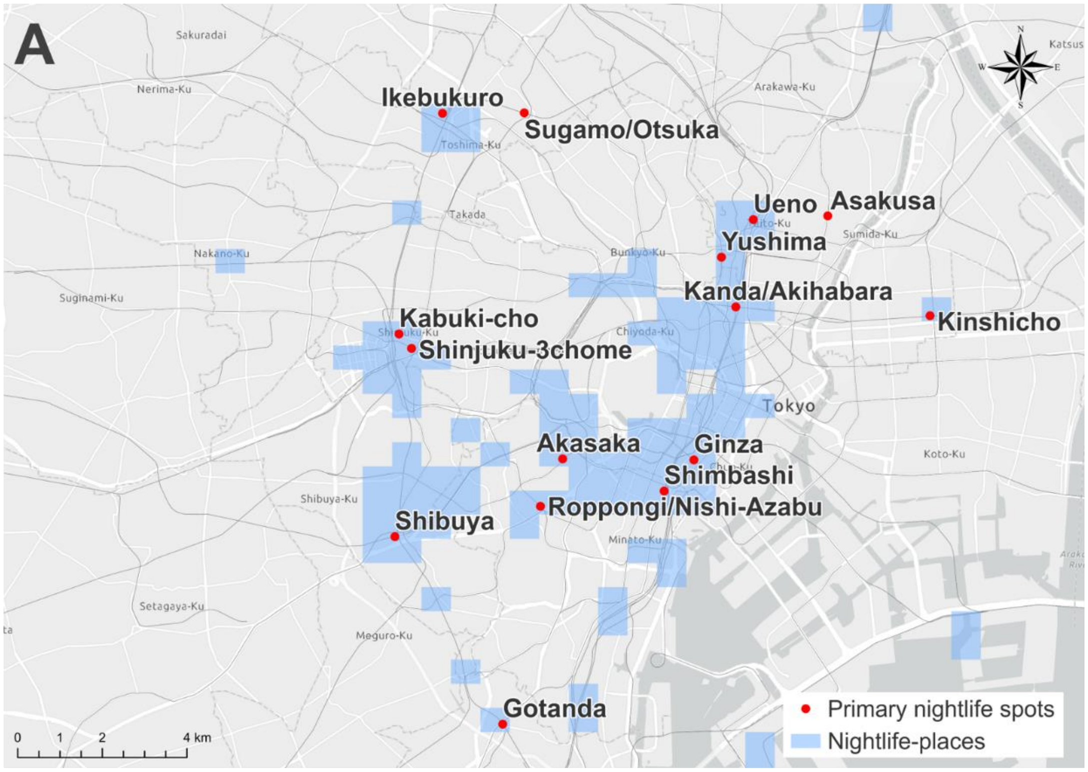
A
Ikebukuro
Sugamo/Otsuka
Ueno
Asakusa
Yushima
Kanda/Akihabara
Kinshicho
Kabuki-cho
Shinjuku-3chome
Tokyo
Akasaka
Ginza
Shimbashi
Roppongi/Nishi-Azabu
Shibuya
Gotanda
Sakuradai
Nerima-Ku
Toshima-Ku
Arakawa-Ku
Takada
Nakano-Ku
Bunkyo-Ku
Taito-Ku
Sumida-Ku
Suginami-Ku
Shinjuku-Ku
Chiyoda-Ku
Chuo-Ku
Koto-Ku
Shibuya-Ku
Minato-Ku
Setagaya-Ku
Meguro-Ku
Primary nightlife spots
Nightlife-places
0 1 2 4 km

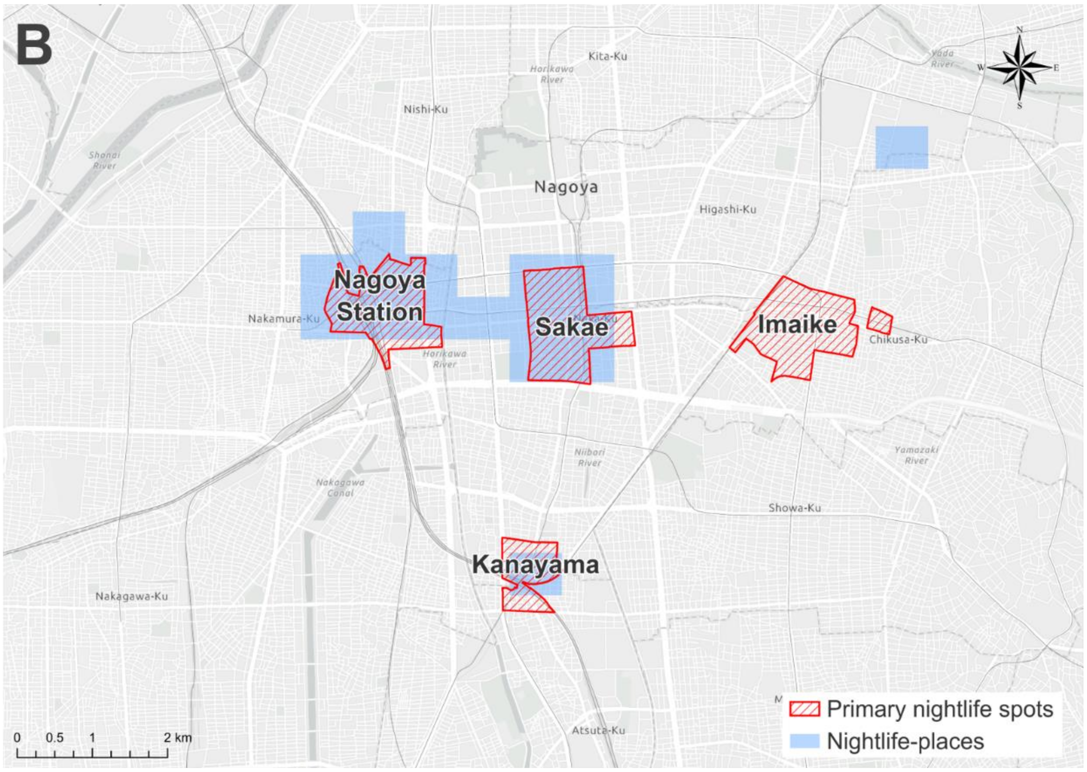
B
Kita-Ku
Horikawa River
Nishi-Ku
Shonai River
Nagoya
Higashi-Ku
Nagoya Station
Nakamura-Ku
Sakae
Imaike
Chikusa-Ku
Horikawa River
Niibori River
Yamazaki River
Nakagawa Canal
Showa-Ku
Kanayama
Nakagawa-Ku
Atsuta-Ku
Primary nightlife spots
Nightlife-places
0 0.5 1 2 km

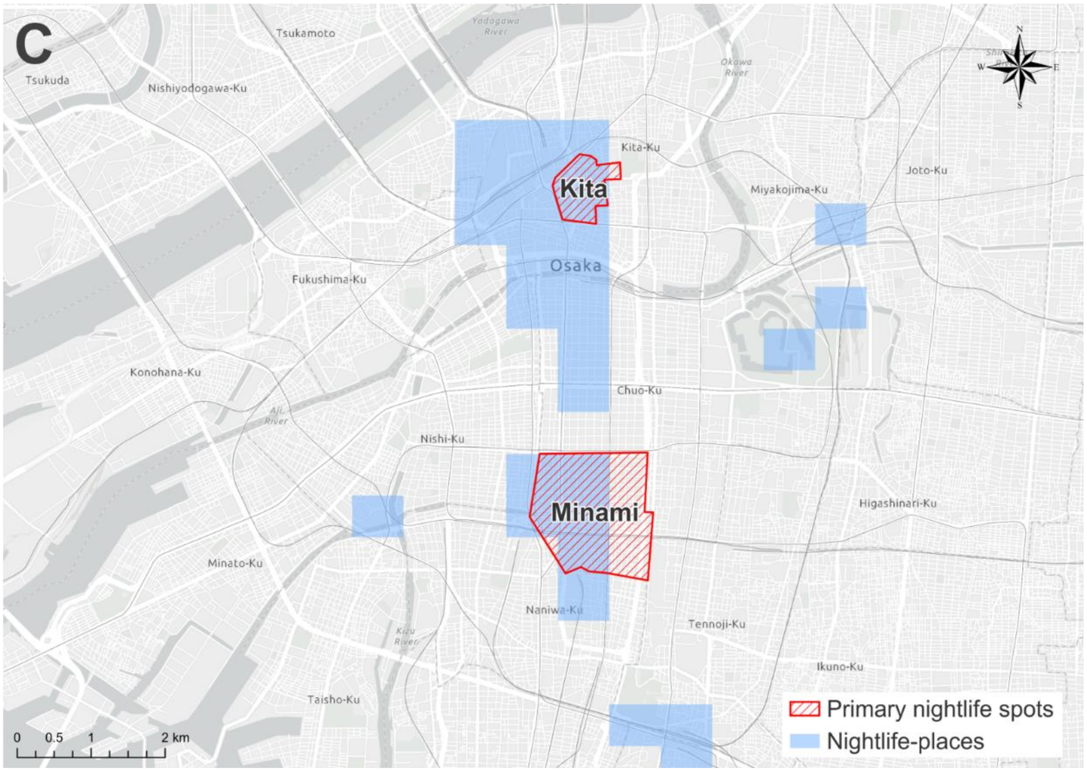

eMaterials Figure 2. Geographical distribution of the primary nightlife spots and nightlife places in Central Tokyo (A), Nagoya city (B), and Osaka city (C). Solid blue areas represent the nightlife places we defined. Red points or red-hatched areas represent the primary nightlife spots listed by the police department websites. Map sources: Esri, HERE, Garmin, FAO, NOAA, USGS, (c) OpenStreetMap contributors, and the GIS User Community.

Although the distribution of business districts/nightlife spots and the workplaces/nightlife-places that we used for our study overlap in many parts, there are a few gaps between them. Since we identified these specific places based on the ratio of the daytime/nighttime population to midnight population, the workplaces or nightlife places did not include the business districts or nightlife spots where large populations reside. However, we considered areas with mixed land-use composing both business districts or nightlife spots and

residential area as inappropriate for measuring the mobility changes because the increase in residential population and the decrease in visitor population in daytime/nighttime often occur simultaneously in such areas. Therefore, the definition of workplaces/nightlife places based on the population differences between midnight and daytime/nighttime should be adequately considered for analyzing mobility changes.

REFERENCES

1. Metropolitan Police Department. The primary nightlife spots in Tokyo. https://www.keishicho.metro.tokyo.jp/kurashi/anzen/sakaribasogo/taisaku.html; Accessed November 19, 2020. (in Japanese)
2. Aichi Prefectural Police Department. An outline of comprehensive measures for nightlife spots safety. http://www.som.pref.aichi.jp/d2w_reiki/430994490107000000000/430994490107000000000/430994490107000000000_j.html; Accessed November 19, 2020. (in Japanese)
3. Osaka Prefectural Police Department. Comprehensive measures for nightlife spots safety –About focused area–. https://www.police.pref.osaka.lg.jp/seikatsu/anzen/8/4106.html; Accessed November 19, 2020. (in Japanese)
